# Cell-Free and Cell-Based Approaches to Explore the Roles of Host Membranes and Lipids in the Formation of Viral Replication Compartment Induced by Tombusviruses

**DOI:** 10.3390/v8030068

**Published:** 2016-03-03

**Authors:** Peter D. Nagy, Judit Pogany, Kai Xu

**Affiliations:** Department of Plant Pathology, University of Kentucky, Lexington, KY 40546, USA; jpoga2@uky.edu (J.P.); kai.xu@uky.edu (K.X.)

**Keywords:** viral replicase complex, phospholipids, sterol, lipid metabolism, lipidomics, virus infection, yeast, *in vitro* replication, membrane contact site, lipid transfer proteins

## Abstract

Plant positive strand RNA viruses are intracellular infectious agents that take advantage of cellular lipids and membranes to support replication and protect viral RNA from degradation by host antiviral responses. In this review, we discuss how *Tomato bushy stunt virus* (TBSV) co-opts lipid transfer proteins and modulates lipid metabolism and transport to facilitate the assembly of the membrane-bound viral replicase complexes within intricate replication compartments. Identification and characterization of the proviral roles of specific lipids and proteins involved in lipid metabolism based on results from yeast (*Saccharomyces cerevisiae*) model host and cell-free approaches are discussed. The review also highlights the advantage of using liposomes with chemically defined composition to identify specific lipids required for TBSV replication. Remarkably, all the known steps in TBSV replication are dependent on cellular lipids and co-opted membranes.

## 1. Introduction

An intriguing aspect of (+)RNA virus replication is the close association of viral replicase complexes (VRCs) with subcellular membranes in plant or animal cells. The membranous structures help sequestering viral proteins, viral RNA and co-opted host factors in confined areas leading to high local concentrations of those compounds for efficient VRC assembly and robust viral RNA replication. The membranes also serve as platforms for VRC assembly and facilitate the activation of viral RNA-dependent RNA polymerase (RdRp). Moreover, the membranous structures help the virus evade cellular defense mechanisms and also protect the viral RNA from degradation. This is facilitated by virus-orchestrated membrane deformations leading to generation of spherules, which are vesicle-like membrane invaginations with narrow openings towards the cytosol, or single- and double-membrane vesicles that harbor VRCs [1,2]. The emerging picture from recent studies with several viruses is that viral proteins and co-opted cellular proteins in combination with cellular lipids play major roles in VRC formation.

Cellular membranes are built from phospholipid bilayers, but also contain other types of lipids and large number of proteins [3,4]. Phospholipids contain a polar head group and long hydrophobic chains that are buried in the membrane bilayer. Various phospholipids modify the physical features of subcellular membranes, such as fluidity and thickness of organellar membranes, and they also affect membrane curvature and charge. In addition to phospholipids, the cellular membranes contain sterols and other lipids. In most eukaryotic cells and organisms, phospholipid synthesis and distribution is tightly regulated, thus making it challenging to modify phospholipid composition of various subcellular membranes using genetic approaches. However, yeast with well-defined lipid metabolism serves as an outstanding model to dissect the role of various lipids in plant (+)RNA virus replication, as described below. Therefore, among the hottest topics in plant virology include approaches to gain deeper insights into the interaction of viral replication proteins with subcellular membranes and lipids and the virus-driven modification of lipid metabolism and transport. In this article, we will discuss the recent progress on dissecting the complex interactions between the virus and the host cell lipids and membranes based on the development of yeast as a model host for TBSV.

## 2. Formation of Membranous Viral Replication Compartments

Different plant (+)RNA viruses select different subcellular compartments or organellar membranes for their replication. Is there a special lipid composition of subcellular membranes favored by different viruses for VRC assembly? Do viruses target preexisting membranes (those made prior to virus infection) or do viruses induce extensive reorganization and/or proliferation of membranes to make them favorable for VRC assembly? Although we do not yet know the answers, new insights have been gained with a small number of plant viruses. For example, TBSV induces extensive membrane proliferation, leading to the formation of multivesicular body-like structures formed mainly from peroxisomes and possibly other organellar membranes, too [5,6,7]. TBSV facilitates this process via using membrane-targeting signals in p92^pol^ and p33 replication proteins, which also have two transmembrane domains. Similar to TBSV, the closely related tombusviruses, such as *Cucumber necrosis virus* (CNV) and *Cymbidium ringspot virus*, also replicate on the peroxisome boundary membrane [7,8,9,10,11], while another tombusvirus, *Carnation Italian ringspot virus* (CIRV) replicates on the outer mitochondrial membrane [12,13,14]. However, tombusviruses utilize alternative subcellular membranes in yeast with altered genetic background or under cell-free conditions [12,15,16], suggesting that selection of subcellular membranes by tombusviruses is flexible to some degree. Other plant viruses target various subcellular membranes, such as endoplasmic reticulum (ER), chloroplast, or vacuolar membranes for replication [1,17,18]. In spite of the different locations of replication compartments, the emerging evidence is that these replication organelle-like structures play comparable roles in replication of various plant RNA viruses.

The formation of spherule-like structures has been studied with several plant (+)RNA viruses, including *Tomato bushy stunt virus* (TBSV), *Brome mosaic virus* (BMV), *Melon necrotic spot virus* and *Beet* *black scorch virus* [1,19,20,21]. Interestingly, both TBSV and BMV usurp cellular membrane remodeling proteins, including the endosomal sorting complex required for transport (ESCRT) machinery [6,22,23,24]. The sequential recruitment of ESCRT protein complexes facilitate bending the membrane towards the lumen of membranous organelles due to inducing negative curvatures in the membrane bilayer. The BMV replicase complex formation also depends on additional membrane shaping proteins, called reticulons [25]. However, usurping membrane-deforming proteins is not enough for spherule formation, but the lipid composition of subcellular membranes used for VRC formation is also critical, as discussed below.

## 3. Approaches to Identify and Characterize Co-Opted Lipids and Membranes Required for Replication of Plant Positive-Sense RNA Viruses

Plant viruses are amazing intracellular agents that can vastly reorganize the subcellular membranes and re-wire cellular lipid metabolism of the infected host cells, forcing them to make sophisticated and elaborate replication compartments [18]. In spite of the daunting task to identify all these complex interactions between the virus and the host cell, rapid progress has been made in recent years due to the development of yeast as a model host for TBSV, CIRV and BMV [26,27,28,29] and the adaption of powerful “OMIC” technologies.

### 3.1. Genome-Wide and Proteome-Wide Approaches

Identification of host membrane proteins or lipids involved in plant virus replication might be achieved through systematic genome-wide screens. However, unlike in the cases of several animal viruses, there is no systematic genome-wide screen yet published with plant RNA viruses in plant hosts. In spite of this, plant virus-host interaction studies have been performed using yeast model host based on available yeast genomic libraries. The high-throughput screens using the single-gene knockout and the essential gene libraries were conducted with BMV and TBSV that led to the identification of over 100 yeast genes affecting either BMV or TBSV replication, respectively [30,31,32,33,34]. Additional yeast-based screens with TBSV, using temperature-sensitive (ts) mutant library and high-throughput over-expression of ~5500 yeast genes in wt yeast contributed to the identification of ~250 more host proteins that could affect TBSV replication [34,35,36,37].

Overall, the genome-wide screens have led to the identification of a dozen host genes involved in lipid biosynthesis, lipid metabolism and intracellular transport [30,31,32]. For example, deletion of yeast genes involved in sterol or phospholipid biosynthesis greatly hinders TBSV replication [38,39]. Among these, the best characterized host genes for TBSV replication are those involved in sterol biosynthesis, such as *ERG4* and *ERG25* (Table 1), and in phospholipid biosynthesis. The latter group of host genes include *INO2*, *INO4*, *OPI1*, and *SCS2* (Table 1). Another interesting example is the stimulatory effect on TBSV replication by the deletion of the yeast *PAH1* gene, which codes for a phosphatidate phosphatase (PAP), the ortholog of the mammalian lipin genes. Pah1p dephosphorylates phosphatidic acid (PA), yielding diacylglycerol (DAG) and triacylglycerol (TAG) storage lipids. In the absence of *PAH1*, the ER membranes expand considerably and the total phospholipid content of the cell increases, which facilitates robust TBSV replication [15]. Interestingly, a major fraction of the TBSV VRCs assembles on the expanded ER membranes in *pah1∆* yeast, suggesting that TBSV could readily utilize ER membranes under certain conditions [15].

Global proteomic-based screens with yeast protein arrays in combination with yeast membrane-based two-hybrid assay (MYTH) with yeast cDNA libraries have led to the identification of over 100 yeast proteins interacting with tombusvirus p33 or p92^pol^ replication proteins [40,41,42]. These approaches have identified ~10 cellular proteins involved in lipid metabolism. The notable proteins identified include Faa3 long chain fatty acyl-coA synthetase, the Scs2 VAP protein, and Fox2 involved in peroxisomal fatty-acid beta oxidation. Altogether, these systems level approaches have revealed exciting new roles of lipid metabolism and transport proteins in TBSV replication [27,43,44].

### 3.2. Lipidomics-Based Approaches

Another “OMICS” approach used to identify critical lipids for tombusvirus replication is based on lipidomics through comparing the lipid composition of cells infected with viruses and the uninfected control cells. Accordingly, yeast and plant cells replicating TBSV showed 38% and 21%, respectively, increase in overall phospholipid content [45], suggesting that TBSV induces new phospholipid biosynthesis. Among the various phospholipid species, PE (phosphatidylethanolamine) level is increased remarkably in both yeast and plant cells supporting TBSV replication in comparison with the virus-free hosts, which is in agreement for the high demand of PE during the formation of TBSV VRCs [45]. An interesting feature of PE is that it promotes negative membrane curvature that could be beneficial during spherule formation [3].

Lipidomics analysis also showed that the PE level was 2.5-fold higher in *cho2∆* yeast than in WT yeast replicating TBSV [45]. Cho2p is a PE methyltransferase that is involved in conversion of PE to PC. TBSV replication increased by ~10-fold in yeast in the absence of Cho2p, further supporting the critical function of PE in TBSV replication and the dependence of TBSV on PE level in membranes.

### 3.3. Transcriptomic Analysis

Virus-induced altered lipid biosynthesis was also measured indirectly through testing mRNA transcript levels for critical phospholipid biosynthesis genes in yeast replicating TBSV. Interestingly, TBSV replication induces the upregulation of phospholipid biosynthesis genes, including *INO1*, *OPI3* and *CHO1*, leading to membrane proliferation in yeast [46]. The TBSV-induced generation of new membranes is achieved in yeast through interaction of the tombusvirus p33 replication protein with the yeast Opi1 FFAT domain protein and Scs2 (a VAP homolog) proteins [46]. These are phospholipid sensors and Opi1 represses the transcription of phospholipid biosynthesis genes [47]. Binding of p33 to Opi1 and Scs2 in the ER membrane prevents Opi1 to enter the nucleus and suppress the expression of phospholipid genes. Accordingly, deletion of *OPI1* transcription repressor in yeast has a stimulatory effect on TBSV replication and also enhanced tombusvirus replicase activity *in vitro* [46]. Altogether, TBSV can reprogram the cellular phospholipid biosynthesis pathway to facilitate its replication in yeast cells.

### 3.4. Cell Biology-Based Approaches

If membranes and lipids are so important for (+)RNA virus replication, then how can viruses usurp those membranes and lipids? Microscopy-based approaches have shown that the TBSV p33 and the CIRV p36 replication proteins target peroxisomal and mitochondrial membranes, respectively, through interactions with cellular proteins, such as Pex19 and the mitochondrial translocase TOM complex [7,8,48]. Since the limiting membranes of these subcellular organelles might not be the most suitable for tombusvirus replication, TBSV induces the enrichment of sterols and PE at the replication sites [45,49]. While the mechanism of p33-driven PE enrichment at the replication sites has not been revealed, sterols are enriched through co-opting cytosolic lipid-binding proteins to the VRC-forming membranous compartments with the help of the p33 replication protein, which binds oxysterol binding protein related proteins (ORPs or OSBP in mammals) and VAP proteins in yeast and in plants [49]. The cellular VAPs and ORPs are present at membrane contact sites (MCS), where subcellular membranes are juxtaposed, thus favoring sterol transfers [50]. The current model predicts that the co-opted ORPs deliver sterols from the ER to the acceptor membranes at MCSs to increase sterol concentrations locally and to stabilize membrane deformation during VRC formation [49].

### 3.5. Cell-Free Studies

Interactions between viruses and hosts, including RNA, protein and lipid interactions, are very complex and a major challenge in cell-based assays. Development of well-defined *in vitro* approaches has been useful to characterize the roles of various components in VRC activities and give mechanistic insights into RNA virus replication. Accordingly, development of four distinct *in vitro* assays with TBSV has tremendously helped our understanding of the roles of membranes and lipids in VRC functions, as discussed below.

*Yeast cell-free extracts*: A powerful approach to dissect the functions of lipids and cellular membranes is based on yeast cell-free extracts (CFE). The CFE preparations can be used to assemble *in vitro* membrane-bound TBSV or CIRV replicases, which support one complete cycle of replication of the viral RNA. The CFE preparations are programmed with the viral (+)RNA template and the purified recombinant TBSV or CIRV replication proteins. The reconstituted CFE-based assay includes all the known replication steps, which could be studied separately, including viral RNA template recruitment, replicase assembly, RdRp activation, (−)RNA and (+)RNA synthesis [28,51,52]. CFEs prepared from yeasts with different genetic background can be used to dissect the functions of various lipids and subcellular pathways in tombusvirus replication. Moreover, the CFEs prepared from mutant yeast strains can be complemented with purified recombinant proteins or artificial lipids added to the *in vitro* reaction.

The membrane fraction of CFE prepared from *cho2∆* yeast supported ~3-fold higher level of TBSV replication than comparable membrane fraction of CFE from wt yeast, demonstrating that increased PE level in cellular membranes is stimulatory to tombusvirus replication [45]. In addition, membrane fraction with depleted PE content prepared from *psd1/psd2/dpl1∆* yeast (genes involved in PE synthesis in yeast) supported ~8-fold less TBSV replication in the *in vitro* replicase reconstitution assay than membrane fraction from the wt yeast [45]. Since all these *in vitro* replicase reconstitution assays contained purified recombinant replication proteins and the soluble host proteins from wt yeast CFE, the results strongly support the direct role of PE in tombusvirus replicase assembly and function.

*Isolated subcellular organelles*: To define the cellular membranes utilized by TBSV and CIRV in the CFE-based assays, ER, mitochondria and peroxisomes were isolated from yeast, followed by VRC reconstitution assay. Interestingly, TBSV could utilize the purified ER membrane most efficiently, though it also replicated in the mitochondrial fraction in the presence of the soluble fraction of yeast CFE [12]. On the other hand, CIRV replicated efficiently in the mitochondrial fraction, and poorly in the isolated ER membrane *in vitro* [12,45]. Importantly, tombusvirus replication in the isolated organellar membranes became insensitive to ribonucleases to some degree, suggesting VRC formation under the *in vitro* conditions. Thus, tombusviruses could usurp intracellular organellar membranes for a full cycle of RNA synthesis *in vitro*. However, these findings do not exclude the possibility that the lipid composition of intracellular organellar membranes are modified during tombusvirus replication in infected cells.

**Figure 1:**
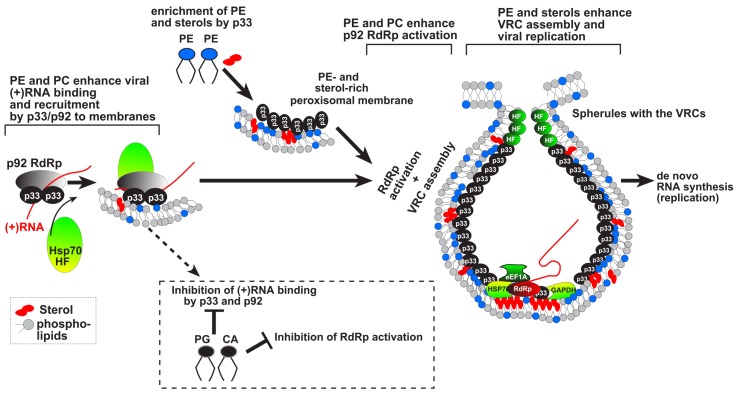
Stimulatory versus inhibitory roles of various cellular lipids in TBSV replication. While neutral phospholipids and sterols stimulate the assembly of the membrane-bound tombusvirus VRCs, other lipids could strongly inhibit virus replication. See further details in the text.

*Stimulation of replicase activation in vitro*: An interesting feature of the tombusvirus RNA-dependent RNA polymerase (RdRp) is that it requires an activation step after its translation to become functional in a membrane-bound form [9,53]. An *in vitro* activation assay was developed for the TBSV p92^pol^ that demonstrated the need for the auxiliary p33 replication protein, two *cis*-acting elements in the viral (+)RNA template and the co-opted heat shock protein 70 (Hsp70) in combination with cellular membranes [9,53,54,55]. Subsequent detailed analysis of the roles of various phospholipids in p92^pol^ activation has revealed the stimulatory function of PE and PC (phosphatidylcholine) on the *in vitro* RdRp activity, while PG (phosphatidylglycerol) showed a dominant inhibitory effect on RdRp activation and binding of p92^pol^ to the viral (+)RNA [54]. These results suggest that the phospholipid composition around the TBSV p92^pol^ affects RdRp activity. Thus, the accessibility of various phospholipids in the targeted membranes might be an important regulatory mechanism for new VRC assembly during the course of tombusvirus infection (Figure 1).

*Artificial lipid vesicles*: Artificial lipid vesicles (liposomes) with known lipid context could be a powerful approach to define the roles of various lipids in virus replication. The reconstitution of active TBSV VRCs in different artificial vesicles has revealed the requirement of high concentrations of PE (more than 70% of total phospholipids) [45]. Interestingly, PE vesicles in combination with the soluble fraction of yeast CFE (which provides soluble host factors) could support the full cycle of TBSV replication, resulting in both (−) and (+)RNA products, the latter in excess amounts. This asymmetrical replication of TBSV in artificial PE vesicles recaptures one of the hallmarks of (+)RNA virus replication. However, the viral RNAs are poorly protected in artificial vesicles, in contrast with the better protection observed with CFEs [45], suggesting that the VRC assembly requires additional protein or lipid factors not present in the artificial PE vesicle assays. *In vitro* experiments with artificial vesicles also demonstrated that the activity of the tombusvirus replicase was stimulated by the addition of 10% PC or 10%-to-30% sterols [45,49], indicating that the complex lipid microenvironment with high PE content is more suitable for TBSV VRC assembly than lipid bilayers containing only PE. Altogether, the artificial vesicle-based *in vitro* assay has unambiguously demonstrated the requirement of PE in tombusvirus replication and VRC assembly [56].

## 4. Additional Plant (+)RNA Viruses

The essential role of phospholipids has also been shown in case of *Red clover necrotic mosaic virus* (RCNMV), which belongs to the Tombusviridae family and it is distantly related to TBSV. The replication protein of RCNMV recruits cellular phospholipase D, which converts PC and PE to phosphatidic acid (PA), into the VRCs [57]. This leads to increased PA levels, which might affect VRC assembly or enhance the activity of the RCNMV RdRp. Unlike the proviral function of PA, addition of PC or PE to culture media of plant cells did not enhance RCNMV replication [57]. The findings that TBSV and RCNMV usurp different species of phospholipids indicate that different viruses could exploit different enzymes, lipids and pathways to build functional VRCs for robust viral RNA synthesis.

Another plant RNA virus for which the roles of cellular lipids have been analyzed is *Brome mosaic virus* (BMV). BMV replication was shown to depend on unsaturated fatty acids made by the cellular ∆9 fatty acid desaturase [58]. In addition, the BMV-induced spherule formation required for replication is affected by long-chain fatty acyl-CoA bound by Acb1 [59]. Deletion of *ACB1* has led to reduced rate of BMV replication and the formation of smaller, but abundant spherules. BMV replication can be complemented by added lipids to the growth media, suggesting that the lipid composition of the cell is critical for BMV replication. A lipidomic analysis of yeast and barley cells replicating BMV revealed close to 30% increase in phospholipid content, suggesting that BMV induced phospholipid synthesis [60]. In addition, PC was enriched and co-localized with the 1a replication protein at the replication sites (perinuclear ER membrane). Moreover, Zhang and colleagues found that 1a interacted with Cho2 methyltransferase, which is inlvolved in PC synthesis. In contrast with the results obtained with TBSV, BMV replication was decreased in *cho2∆* yeast [45], suggesting that the phospholipid dependence of these two unrelated viruses differ [60].

## 5. Conclusions

Recent discoveries using live yeast and plant cells and cell-free assays revealed major roles for cellular lipids and membranes in TBSV VRC assembly [61,62]. First, the subcellular membrane (peroxisome or ER) serves as a pre-assembly platform for protein-RNA complexes, including p33 and p92^pol^ replication proteins, the viral (+)RNA and co-opted host factors. The second process is the virus-induced enrichment of PE and sterols at the membranous sites of replication. Third process is the VRC assembly, which is driven by interactions between p33 replication protein, membrane-bending proteins, such as the co-opted cellular ESCRT proteins, and mostly PE and other phospholipids and sterols in subcellular membranes. These interactions lead to deformation of membranes around the replicase complex. Another process affected by lipids is the activation of the RdRp function of p92^pol^ replication protein within the membrane-bound VRC. In addition to *cis*-acting elements in the TBSV (+)RNA, the p33 replication co-factor as well as cellular co-factors such as heat shock protein (Hsp70), the activation of p92^pol^ replication protein is enhanced by neutral lipids in the host cell membrane [53,54]. Thus, all known steps in TBSV VRC assembly are dependent on co-opted cellular lipids and membranes. The complex interplay between TBSV and cellular lipids and membranes is unlikely unique, and future studies with many (+)RNA viruses will uncover intriguing interactions involving co-opted lipids that could lead to antiviral targets and novel therapies.

## Figures and Tables

**Table 1:** Identified lipid metabolism or lipid transport proteins involved in TBSV replication.

Gene	Cellular Function	Interaction
*CHO2*	Phosphatidylethanolamine methyltransferase (PEMT)	
*ERG4*	delta24 (24-1) sterol reductase	
*ERG25*	ergosterol biosynthesis	
*ERG10*	Acetyl-CoA C-acetyltransferase, mevalonate and sterol biosynthesis	
*FAS2*	Alpha subunit of fatty acid synthetase	
*FEN1*	Fatty acid elongase, involved in sphingolipid biosynthesis	
*FOX2*	peroxisomal fatty acid beta-oxidation pathway	p33/vRNA
*GPT2*	Glycerol-3-phosphate acyltransferase, involved in lipid biosynthesis	
*INO2*	Transcription factor; required for derepression of phospholipid biosynthetic genes	
*INO4*	Transcription factor; required for derepression of phospholipid biosynthetic genes	
*MCT1*	S-malonyltransferase/fatty acid metabolism	
*OLE1*	Fatty acid desaturase, required for monounsaturated fatty acid synthesis	
*OPI1*	Transcriptional regulator, function in negative regulation of phospholipid biosynthetic genes	p33
*OSH3*	Member of an oxysterol-binding protein family, function in sterol metabolism	p33
*OSH5*	Member of an oxysterol-binding protein family, function in sterol metabolism	p33
*OSH6*	Member of an oxysterol-binding protein family, function in sterol metabolism	p33
*OSH7*	Member of an oxysterol-binding protein family, function in sterol metabolism	p33
*PAH1*	phosphatidate (PA) phosphatase; dephosphorylates PA to yield diacylglycerol	
*POX1*	Fatty-acyl coenzyme A oxidase, fatty acid beta-oxidation pathway in the peroxisomes	
*SCS2*	VAP homolog, ER-PM contact site, regulates phospholipid biosynthesis	p33
*SCS22*	VAP homolog, regulates phospholipid biosynthesis	
*TGL2*	triacylglycerol lipase/lipid metabolism	

The functions of yeast genes (shaded) have been characterized in detail in TBSV replication.
